# Context-Sensitive Ecological Momentary Assessment: Application of User-Centered Design for Improving User Satisfaction and Engagement During Self-Report

**DOI:** 10.2196/10894

**Published:** 2019-04-03

**Authors:** Preethi Srinivas, Kunal Bodke, Susan Ofner, NiCole R Keith, Wanzhu Tu, Daniel O Clark

**Affiliations:** 1 Indiana University Center for Aging Research Indianapolis, IN United States; 2 Regenstrief Institute Indianapolis, IN United States; 3 Indiana University School of Medicine Department of Biostatistics Indianapolis, IN United States; 4 Indiana University School of Health and Human Sciences Department of Kinesiology Indianapolis, IN United States; 5 Indiana University School of Medicine Department of Medicine Indianapolis, IN United States

**Keywords:** mhealth, health status, obesity, ecological momentary assessment

## Abstract

**Background:**

Ecological momentary assessment (EMA) can be a useful tool for collecting real-time behavioral data in studies of health and health behavior. However, EMA administered through mobile technology can be burdensome, and it tends to suffer from suboptimal user engagement, particularly in low health-literacy populations.

**Objective:**

This study aimed to report a case study involving the design and evaluation of a mobile EMA tool that supports context-sensitive EMA-reporting of location and social situations accompanying eating and sedentary behavior.

**Methods:**

An iterative, user-centered design process with obese, middle-aged women seeking care in a safety-net health system was used to identify the preferred format of self-report measures and the look, feel, and interaction of the mobile EMA tool. A single-arm feasibility field trial with 21 participants receiving 12 prompts each day for momentary self-reports over a 4-week period (336 total prompts per participant) was used to determine user satisfaction with interface quality and user engagement, operationalized as response rate. A second trial among 38 different participants randomized to receive or not to receive a feature designed to improve engagement was conducted.

**Results:**

The feasibility trial results showed high interface satisfaction and engagement, with an average response rate of 50% over 4 weeks. Qualitative feedback pointed to the need for auditory alerts. We settled on 3 alerts at 10-min intervals to accompany each EMA-reporting prompt. The second trial testing this feature showed a statistically significant increase in the response rate between participants randomized to receive repeat auditory alerts versus those who were not (60% vs 40%).

**Conclusions:**

This paper reviews the design research and a set of design constraints that may be considered in the creation of mobile EMA interfaces personalized to users’ preferences. Novel aspects of the study include the involvement of low health-literacy adults in design research, the capture of data on time, place, and social context of eating and sedentary behavior, and reporting prompts tailored to an individual’s location and schedule.

**Trial Registration:**

ClinicalTrials.gov NCT03083964; https://clinicaltrials.gov/ct2/show/NCT03083964

## Introduction

### Background

Precision medicine is an approach to care that involves classifying individuals into subpopulations that differ in their susceptibility to a particular disease or in their response to a specific treatment [1]. Subpopulations can be defined by genetics, but they can also be defined by behavioral and environmental exposures that lead to differential responses to biomedical, behavioral, and environmental interventions. The latter targets might be referred to as precision health interventions and may be advanced by better measures of behavioral and environmental exposures [2]. Environmental exposures can be relatively constant or vary over relatively short intervals of time, which together make up what we will refer to in this study as situations.

Large-scale precision health research efforts are in final planning [3] or just underway [4] and include the measurement of real-world and real-time physiological data from sensors such as accelerometers, heart rate, and glucose monitors, among others. Although detailed physiological measures will help with early detection of changes in physiological states and thus improve prevention or early treatment, precision health will also require measuring the situations and behaviors that directly or indirectly affect physiology [5]. Social, behavioral, and environmental factors contribute as much or more to health and longevity as other major domains including medical care and genetics [6]. Thus, better measures of behavior and situations are the keys to not only understanding behavior but also precision health interventions and ultimately better health and longevity.

Furthermore, 1 of the techniques widely used to obtain a situational or contextual understanding of daily life includes experience-sampling methodology [7]. This method includes self-report measurement but in a form where a person responds to subjective questions multiple times a day. This technique has often been attributed to overcoming methodological problems owing to memory and recall [8,9]. Furthermore, this method has high ecological validity and supports within-subject investigations [10,11]. Previous health research has attempted to execute experience sampling on technology devices such as personal digital assistants and pagers. With advancing technology, experience sampling has also been executed on mobile devices such as mobile phones. Often referred to as ecological momentary assessment (EMA) in health research, EMA is typically completed as persons experience something in their natural environment.

EMA is considered the gold standard of experiential sampling in health research [8]. However, self-report through EMA can be a burden, given the need to administer instruments multiple times in a day. Furthermore, the collected data can suffer from poor adherence and misreporting, especially if the instrument is cumbersome to use or does not suit individually-variable reporting needs and preferences (eg, sleep and work schedules and location triggers) [12]. This motivates a need for sampling tools that not only support situation-dependent, real-time self-report multiple times in a day but also are (1) low burden, (2) supportive of recurrent use, and (3) tailored to users’ needs.

### Objectives

This paper addresses these needs with a case study involving self-report measurement of location and social situations accompanying eating or sedentary behavior. This work was carried out in the context of a randomized trial among middle-aged, obese women cared for in a safety-net health system (NCT03083964) [13]. We report here the design and implementation of self-report measures of eating and movement behavior specific to users’ location and social contexts. Each measure was developed through an iterative, user-centered design process involving obese, middle-aged women and deployed in a field trial to establish usability. The specific contributions of this paper are (1) a series of design constraints identified as important to consider and satisfy when designing mobile EMA interfaces, which are personalized to users’ preferences, (2) 6 refined measures for self-report of eating and sedentary behavior, specific to location and social context using a mobile device, (3) a characterization of the ways in which individuals prefer to self-report eating and movement, along with perceived benefits and challenges of this self-reporting, and (4) field trials of feasibility with attention to response rates.

## Methods

### Overview

This study used an iterative user-centered research and design approach that comprised 4 phases (Table 1) to support the design and development of a mobile app. All sessions were audio and video recorded. Recordings were reviewed by stakeholders and designers before making design modifications to the EMA system. Qualitative thematic analysis was performed to identify and iteratively refine themes. This included 1 researcher coding and analyzing data using ATLAS.ti 8 Mac (ATLAS.ti). The codes and themes were reviewed, iteratively rectified, and agreed upon in consultation with other researchers on the team. The findings of Phases 1 and 2 were paired with design literature to guide the development of prototypes for evaluation in Phases 3 and 4 field trials. Overall, iterative participatory design and review sessions helped progress the identified measures from low-fidelity paper sketches to high-fidelity prototypes.

**Table 1:** Iterative and participatory user research and design of ecological momentary assessment system.

Phase	Research method	Duration	Stakeholders/users, n
Phase 1: Exploratory ideation	Focus group with stakeholders; 1-1 design session with users	60 min; 4560 min	6 stakeholders; 5 users
Phase 2: In-lab evaluation	Scenario-based think-aloud usability evaluation	4560 min	6 users
Phase 3: Field Trial 1	User evaluation of ecological momentary assessment (EMA) system in the field (feasibility test)	4 weeks	21 users
Phase 4: Field Trial 2	Response rate comparison of 2 versions of EMA system	4 weeks	38 users

### Setting and Users

The target users of the EMA system live within a single city-county area of the Midwest. This study recruited patients aged 35 to 64 years and currently receiving care in 1 of Eskenazi Health’s federally qualified health centers (FQHCs). Eskenazi Health is 1 of the 5 largest safety-net health systems in the nation. FQHCs gave us access to obese, middle-aged women who had had a provider-referral to the Healthy Me program. A primary care provider may refer a patient with a body mass index (BMI) of 30 or higher to meet with a Healthy Me coach. In this clinic-based program, health coaches counsel adult, obese patients and create an action plan for increased physical activity and encourage patients to make healthy food choices with an emphasis on controlling portions of food they consume. Healthy Me coaches are certified in behavior change counseling and fitness instructions, and they are present 2 or more days per week in each of the FQHCs [14].

### Phase 1: Exploratory Design Ideation

Phase 1 involved ideation and designing of self-report measures on the basis of existing literature and user and stakeholder requirements. It included (1) a focus group session with the research team’s primary stakeholders (social scientist, exercise physiologist, visual communication experts, and personal trainers) and (2) a 1-on-1 design session with 5 Healthy Me patients (P1-P5).

#### Methods

The focus group session lasted for an hour and identified the core self-report measures of behavior and situation specific to the project—food and drink consumption, physical movement, users’ location, and social copresence. The focus group session also resulted in the development of 4 questions, each representing a single measure (version 1, V1). Questions in V1 were straightforward and required selection from a list of prepopulated response options. For instance, questions on eating/drinking and social copresence included options *yes* and *no*, whereas questions on location and physical activity included options created by stakeholders from expert knowledge and previous literature.

Following the initial focus group session, we conducted 1-on-1 design sessions with 5 Healthy Me patient volunteers (Participant 1 through Participant 5 or P1 through P5). Participants for this session were identified through announcements by coaches at the end of Healthy Me classes. The 45 to 60 min design session had 2 parts. During the first part, participants were presented with the problem domain and V1 questions created during the focus group session. This was followed by a brainstorming phase, where participants helped identify components for version 2 (V2). Each participant was compensated with a US $25 gift card at the end of a session.

#### Results

The fundamental principles of human-computer interaction informed us in designing an interactive prototype to support a user in responding to V2 EMA questions. As such, differences between V1 and V2 included refined and context-based response choices with images, icons, and a time component to every question to aid users’ recall from within a referenced time frame. Specifically, V2 questions included (1) context-based suggestions for location in addition to search option (eg, prepopulated options to choose from, on the basis of the device’s current location), (2) simplified response choices for physical activity, (3) detailed response choices for eating behavior to help users provide more detailed data (eg, meal or snack as opposed to a *yes*), and (4) detailed response choices for social copresence.

### Phase 2: In-Lab Evaluation Studies

This phase involved evaluation of V2 measures identified in Phase 1.

#### Methods

In this phase, 6 female Healthy Me patients aged 35 to 64 years (50% African American, 50% nonHispanic white), recruited through snowball sampling, evaluated V2 measures. We refer to these participants as P6-P11. Each session lasted 45 to 60 min, where participants qualitatively evaluated and provided feedback on V2 questions delivered through an interactive, mobile prototype. Findings from this session resulted in the development of version 3 (V3) questions.

#### Results

Some of the findings from the in-lab evaluation session included mixed support for image icons placed next to each response option and the need for simplified question and response options. For instance, although some participants found the prototype intuitive, others were confused with associating an image icon with the response option. This led to an executive decision on rolling back or excluding image icons as the immediate project scope was to design a mobile EMA tool supporting the core qualitative findings from Phases 1 and 2. Overall, the differences between V2 and V3 questions included excluding image icons, refined questions with fewer words and a time frame reference, and refined response choices that allowed the user to maintain privacy. Specifically, V3 questions included (1) response choices that helped users select the type of location as opposed to providing the actual physical address, (2) simplified response choices for physical activity that focused on users reporting whether they had walked or not, (3) simplified response choices for eating behavior that direct users to consider everything other than drinking water as an eating event, and (4) means to prepopulate social connections with names or relationships.

### Summary of Findings From Phases 1 and 2

**Figure 1:**
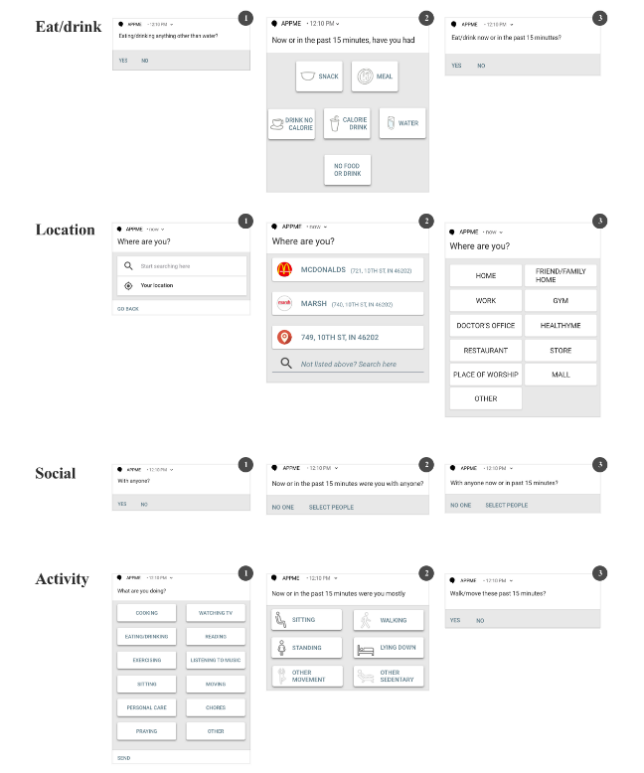
Three versions of ecological momentary assessment questions resulting from iterative design process.

Phase 1 (ideation) and Phase 2 (in-lab evaluation) work produced the 6 qualitative themes below that guided our design decisions on questions and response choices among versions V1, V2, and V3 (Figure 1).

#### First: Privacy

Most participants disliked the idea of sharing their location with a technology solution:

My husband is very paranoid when it comes to technology and often says to me that someone is watching us through it. I would rather not share my exact address with the app.

P3

Participants attributed this feeling toward a lack of trust as to where their data were stored and who had access to them. The following is an example:

I don’t know who is going to look at this data. It is like I am being policed

P1

Such participants suggested reporting an approximate location by selecting names associated with the location’s address:

I don’t mind saying I am at a restaurant as opposed to reporting I am at the McD on the corner of 16thstreet, if you know what I mean.

P11

#### Second: Active or Sedentary

Participants reported difficulties in identifying if they had or had not been moving enough to self-report physical activity while responding to an EMA question:

If I were sitting down but moving my arms up and down, wouldn’t that still count as movement?

P4

A few participants valued and thought an interface would be more intuitive and natural if the question included images depicting the action that was of interest:

It would be easier for me to remember what I was doing when I see a picture of someone performing the activity, like a walking figure.

P2

On the other hand, some participants reported they would be confused if they saw images with actions that represented an unfamiliar action:

I see a picture of someone standing on one leg and it says here other movement. Is this yoga or dancing?Perhaps something else?

P10

Participants felt strongly about the need for a simplified and direct list of options from which to choose when reporting activity:

If all the app wants to know is if I moved or not, why not just ask that as opposed to asking me more details?

P2

#### Third: Reporting Food/Drink Consumption

Participants were very diverse in the ways they thought about and wanted to report food or drink consumption. Although some thought water would fall under food/drink self-report, others considered water being independent of any other food or drink they consumed:

I wouldn’t want to report when I had water to drink. I am drinking water all day. The app would think I am eating or drinking something all day then.

P5

In such cases, participants suggested improving clarity on the question to help remind them not to think about water while self-reporting food/drink consumption:

Maybe the question can be–did you eat or drink something other than water?

P8

Several participants suggested improving their recall ability by including more response options:

It would be more easier if the question listed out options like snack or meal for food and maybe if it was diet or normal drink to help me remember. Yes or no is fine, but it would help if I saw better options.

P6

However, a few other participants felt they would face a challenge identifying and associating a category to whatever they had consumed. Several participants doubted the accuracy of this technique:

I think half a sandwich is a snack, that is just me. But I am not sure if that is a snack for others.

P9

#### Fourth: Who Is Around Me

Participants discussed the need for maintaining the privacy of their social connections while using the app. Some felt strongly about not sharing the name of the person they were spending time with when responding to the EMA questions as they thought they were violating others’ privacy without their consent:

I don’t want to tell some app Sue is with me without her consent.

P4

In such cases, participants suggested means for the app to allow selecting a relationship type as opposed to a person’s name:

Why don’t I say my sister is with me instead?

P4

Several participants reported increased desire and flexibility in prepopulating the names or relationships of most people with whom they interacted. This prepopulated list was used to create response options for the social copresence question. Participants appreciated reporting *other* in instances the response options did not hold a specific name of a person or relationship:

...it is easy to choose other when I don’t see the name of the person who is with me.

P7

#### Fifth: Quick Interaction

All participants strongly preferred selecting a value through a single tap:

I like how I tap and can move onto next question. This is really quick.

P8

That being said, some participants discussed instances when they thought a single tap to move onto next question could be error prone:

This is really quick. But, what would happen if I tapped yes to the eating question by mistake? Can I go back and re-do the selection?

P11

#### Sixth: Time-Based Reporting

Several participants expressed concern toward disruptions at times they prefer not being disturbed (eg, while asleep):

Will I be receiving these messages during the night? I don’t think I can respond. Can I get these questions when I am awake?

P2

Some participants felt strongly about the need to restrict the time frame on the basis of which they recalled before self-reporting:

I think it will make more sense if the app asked me what I did in the past 10 or 15 minutes

P6

Some participants rejected the time-oriented interface and strongly preferred a more relaxed qualitative instrument or something they can tune to their own subjective experience:

It is hard to know what I was doing exactly 15 minutes ago.

P8

These participants explained that the more relaxed the time frame such as since the last time they responded would enable them to be more accurate in remembering from the last time they responded:

I will remember what I said last time to the app.

P9

### Discussion

The 6 qualitative findings from the exploration (described above) and in-lab evaluation phases were coupled with self-report and mobile usability literatures [15-24] to create 5 core design constraints (described below) that guided our design decisions for phases 3 and 4 (Table 2).

#### Design to Support Rapid Recall

A significant portion of Phase 1 and Phase 2 findings included discussing the need to reduce effort during recall. First, participants discussed the need for aiding in rapidly recalling their behavior on the basis of simplified response choices. Second, although the stakeholders discussed the need for capturing responses that represent behavior in the time between 2 EMA prompts, the Healthy Me patients expressed confusion in determining a time window they had to use as reference while performing a recall. Discussions included confusion when users skip/miss EMA prompts and the need for a time window as reference to help recall information. For instance, P1 stated the following:

What would happen if I didn’t respond all day and see something at the end of the day?

P3 stated the following:

Do I respond to the question considering what I was doing at the moment I saw it or am I responding based-off of something in the recent past?

#### Design for Low Effort From User

Self-report can be a burden given the need to respond multiple times a day and while in the midst of daily activities. The need to respond to a notification can force users to interrupt an ongoing activity, consequently leading to increased burden/frustrations. This is true especially when the task of responding is effortful. Given this, it was a critical need for the system to require minimal effort while responding to a question post recall.

**Table 2:** Design decisions on the basis of constraints.

Constraint	Design decision
Design to support rapid recall	Rapid recall can be supported through the provision of a reference time frame where users can perform recall by focusing on the time window.
Design for low effort from user	A system that is capable of sending self-report questions as a group of message notifications can allow users to respond on the go, with a single tap on a mobile device. The burden can be further reduced if the group of messages is dependent on the users’ context. For instance, the EMA^a^ system can skip asking about food/drink consumption if the user is physically located in a restaurant when a self-report message group is sent, or the EMA system can provide suggestions for location on the basis of the device’s location to help users avoid searching for an actual address.
Design to capture situations that accompany a behavior	To ensure a response is captured close to a situation, the EMA system should not allow users to respond after a set number of minutes have passed as it is likely the context of the user changes over time. To support this need, participants suggested a 30-min window for capturing responses and context, that is, a notification with a question disappears from the user's screen if the user does not respond within 30 min of receiving the notification.
Design to capture better quantity and quality of data	To maximize data capture, the EMA system can prompt a response at times when the user is awake and does not want to be disturbed. Furthermore, 1 way to personalize this experience for users is to have an onboarding process where the users can set preferences for when they will typically be available to receive EMA questions. Moreover, 1 way to capture data with improved quality, specifically for eating behavior, is to include response options where users can choose the type of food or drink. Similarly, social copresence can also include detailed response choices, which users can prepopulate with their social connections before EMA usage and select those when prompted for social copresence.
Design for user’s safety	A notification can be held back if the EMA system identifies the user is moving in a vehicle. This avoids putting the user in danger.

^a^EMA: ecological momentary assessment.

#### Design to Capture Situations That Accompany a Behavior

It became clear from the ideation sessions that users are present in dynamically changing contexts of time, space, activity, and social connection. Hence, the EMA system should not only capture self-report measures but also the context or situations that entail particular user responses.

#### Design to Capture Better Quantity and Quality of Data

Both Phase 1 and Phase 2 participants pointed to the need for capturing better quality, in addition to quantity of data. Stakeholders discussed the need for capturing detailed information on social copresence to help identify patterns in eating or movement behaviors specific to social connections. Similarly, several participants from the 1-on-1 design sessions expressed concern about the lack of detail in reporting eating behavior. As such, all participants had differences in perception in determining when they have had something to eat or drink. For instance, P2 stated the following:

If I had a piece of candy do I still report yes to this question? I don’t think that is eating really.

P4 stated the following:

I usually know when I have eaten a meal, which can be a bigger portion size, as opposed to something like a small snack. It would be nice if I can report clearly what I had to eat because otherwise the app is going to think I am eating something all day.

#### Design for Users’ Safety

Participants from both phases raised the issue of impacting user safety in instances the user is required to, but is unable to, respond to message notifications. Hence, there is a need to design for safety and avoid penalizing a user for a nonresponse at unsafe moments (eg, while driving).

#### Ecological Momentary Assessment System: V3-Simple

**Figure 2:**
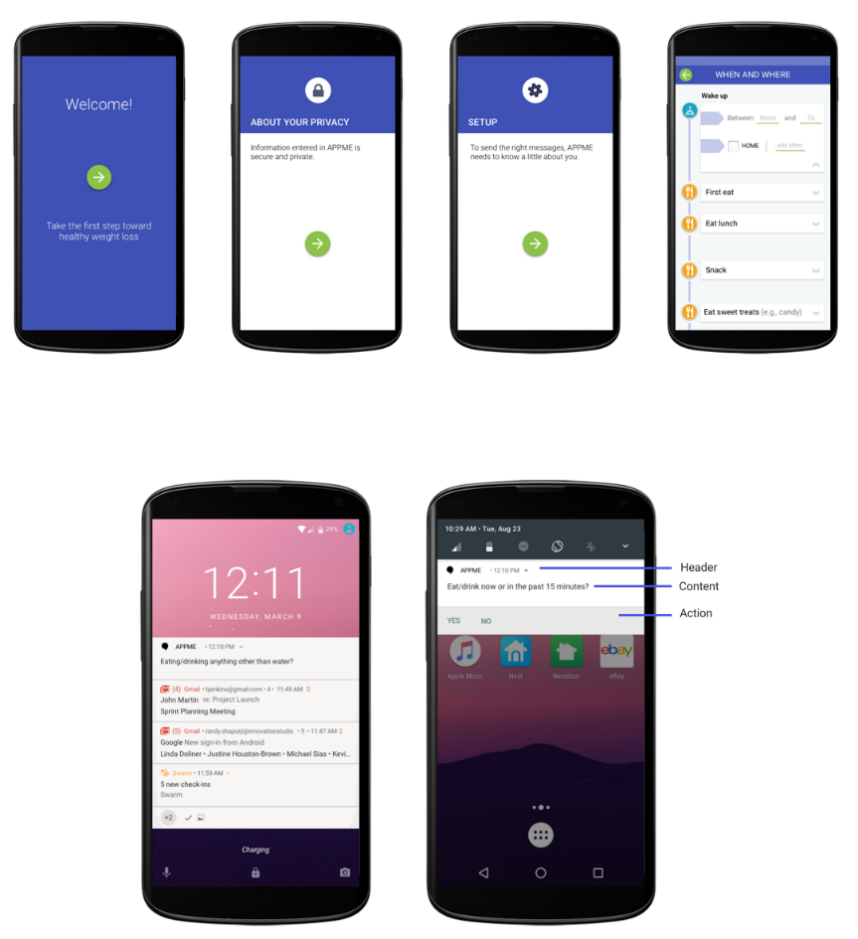
One-time onboarding screens (top) and example ecological momentary assessment question in device’s notification drawer (bottom left) and structure of an example ecological momentary assessment question (bottom right).

From the 6 qualitative findings and 5 design constraints, an interactive and functioning EMA app was designed and developed to run on an Android smartphone. We chose to develop an Android app owing to (1) the availability of reusable Google libraries and services that can help identify the device’s location or movement, (2) access to open-source code in the Android ecosystem that can be reused to save engineering effort, and (3) engineers’ existing knowledge of Android app development practices. Although we expect modifications to the user interaction design, we do not anticipate modifications to the design constraints if this tool were to be deployed in a different smartphone such as an iPhone to suit operating system and hardware needs. To achieve maximum timely awareness and context learning, this system also included an onboarding process (Figure 2).

**Figure 3:**
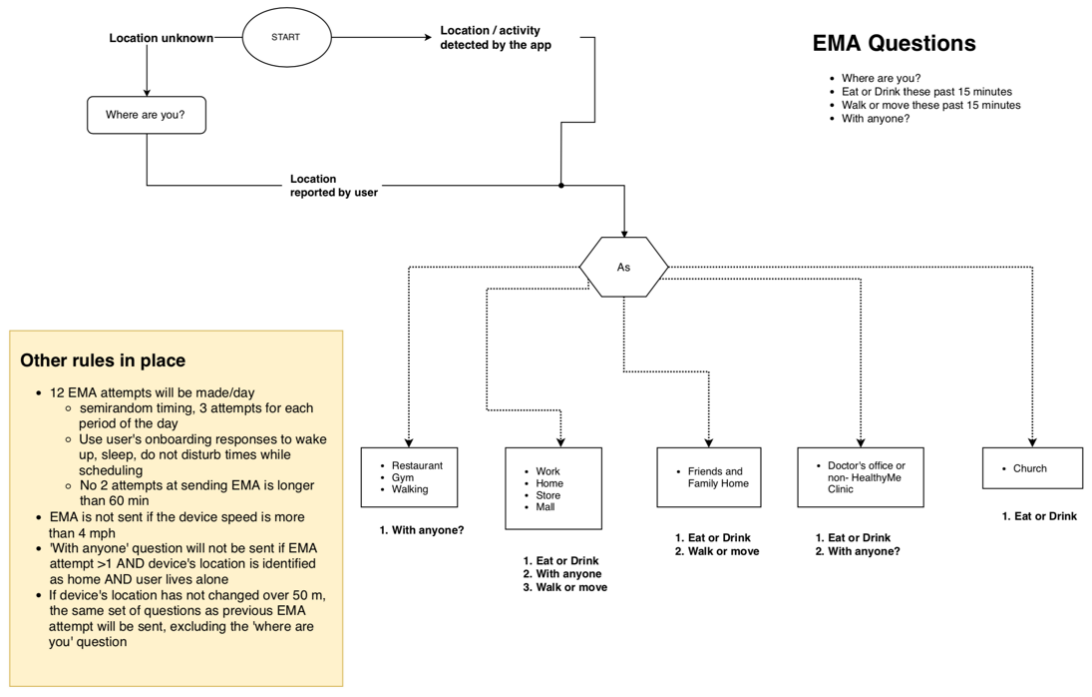
Flowchart depicting the logic used to identify the ecological momentary assessment questions in a group. EMA: ecological momentary assessment .

The onboarding process included users performing a 1-time setup by selecting approximate times when they woke up, ate, slept, and wished to not be disturbed. We envisioned this information to guide the timing of EMA questions for each participant. The EMA system was programmed to begin sending EMA questions the day following the onboarding setup to ensure the complete capture of data in a day. Any EMA question was designed to mimic a notification as identified by Google Material Design and comprised components such as header, content, and action buttons. Furthermore, EMA questions were developed to always appear first in the device’s notification drawer (Figure 2) and included playing a sound clip (device’s default for receiving a notification) every time a question was received on the device. The system was programmed to send EMA questions in groups. Overall, 12-question groups were prescheduled at the beginning of the day for every participant. Each response notification was timed to occur within the waking time and not during sleep or *do not disturb* times as selected by a participant during the onboarding process. By default, each message group comprised the 4 EMA questions regarding location, social presence, eating, and movement behaviors. However, the system removes EMA questions from a group on the basis of the participant’s context, such as device’s movement and location. The logic rules that guide the system and ultimately the user experience are shown in Figure 3.

#### Identifying the Device’s Location and Movement

In general, the system uses a phone’s sensors and Google’s movement and location identification services to identify the device’s movement and location, respectively. For locations not in Google’s service and particularly the participant’s home, the system uses the following approach. The location *Home* is self-labeled by the participant (with help from study staff as needed) during onboarding. This system uses the recorded location coordinates within a 50-meter radius to identify whether or not a device is at the participant’s home. For instances when the system is unable to locate the device, the participant’s response to the location question is used to present appropriate follow-up EMA questions specific to that context. Any location coordinate recorded by the system is anonymized and stored in an encrypted, highly secure study server. This functionality of the system was communicated to all the participants (during the consent process), with participants having the additional option to remove their home location coordinates from the system if they changed their mind during the study period.

### Phase 3: Field Trial 1

This field trial included testing the feasibility of the EMA system (V3-simple), with a focus on participants’ understanding of EMA questions and response choices.

#### Methods

Participants for Phases 3 and 4 were recruited and enrolled following the human subjects’ protection approval and protocols of the larger trial under which this work occurred [13]. This included an incentive of 10 cents for each completed EMA question, up to a maximum value of $10 over 4 weeks. Before the EMA trials, research assistants completed an in-home baseline assessment that captured sociodemographic and BMI data, and the Newest Vital Sign score for health literacy [25] data (see Table 3). During the 4-week trial, 21 Healthy Me patients (P12-P33) received 12 EMA message groups per day, every day on the basis of their onboarding times. The actual EMA questions for each group were determined on the basis of the phone’s context and participant responses to the location question. Each EMA question behaved similar to a short message service notification in a smartphone, with a sound alert every time a question was received on the phone. This field trial included gathering qualitative feedback on the question and response choices, overall satisfaction [26], and perceived interface quality [27]. Table 3 shows the characteristics of participants in Field Trial 1 as well as Field Trial 2.

**Table 3:** Descriptive statistics for participants in field trials 1 and 2.

Participant characteristics		Field Trial 1 (n=21)	Field Trial 2 (n=38)
Age (years), mean (SD)		52.2 (7.6)	52.4 (8.5)
**Race, n (%)**			
	Black or African American	16 (76.2)	33 (89.2)
	White	5 (23.8)	4 (10.8)
Number of households, mean (SD)		2.2 (1.5)	2.8 (1.9)
Household income, mean (SD)		20,480 (23,022)	19,088 (12,771)
**Education level, n (%)**			
	High school	7 (33.3)	1 (2.7)
	College/university	14 (66.7)	22 (59.5)
**Work status, n (%)**			
	Not working	17 (81.0)	14 (37.8)
	Working	4 (19.0)	23 (63.9)
**Hours worked, n (%)**			
	1-10	1 (25.0)	13 (36.1)
	11-20	1 (25.0)	2 (15.4)
	21-30	1 (25.0)	11 (84.6)
	31-40	1 (25.0)	7 (53.8)
**Shift work, n (%)**			
	First shift (6-8 am)	2 (50.0)	7 (53.8)
	Second shift (2-5 pm)	—^a^	3 (23.1)
	Varies	2 (50.0)	3 (23.1)
Weight (lbs), mean (SD)		257.0 (57.6)	250.9 (56.8)
Body mass index, mean (SD)		45.5 (8.4)	45.2 (10.7)
**Low health literacy, n (%)**			
	No	11 (52.4)	12 (32.4)
	Yes	10 (47.6)	25 (67.6)

^a^Data not available.

**Figure 4:**
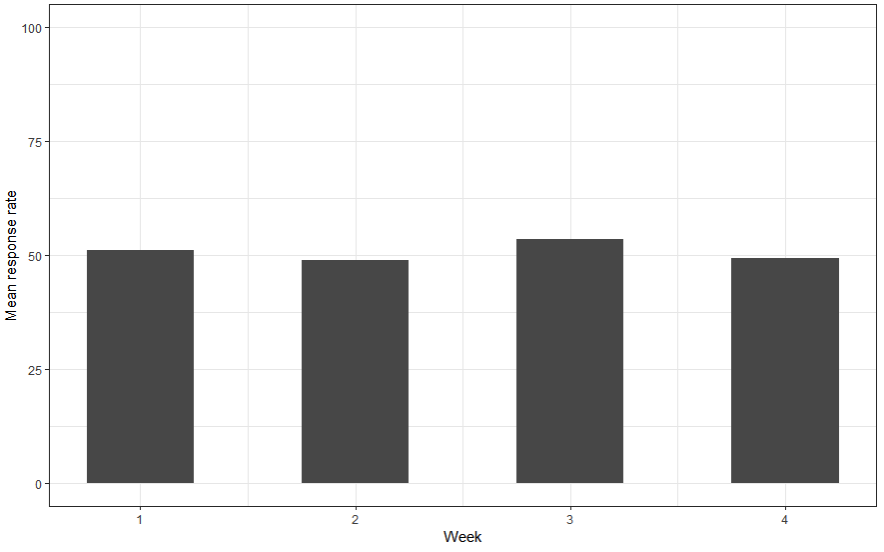
Mean weekly response rates for participants using version 3-simple in Field Trial 1.

#### Results

This trial was also used to determine participants’ response rate to the EMA system. A common finding on reviewing the quantitative data suggested that all participants who began responding to the first question in a message group continued to complete all the questions in that group. Hence, the presence of a response to the first question within an EMA message group was used to calculate a participant's response rate. The overall response formula was: total number of first question responses over the total number of EMA question groups.

On the basis of this formula, the overall average response rate for participants in Field Trial 1 was identified as 50.3%. The mean weekly response rates were consistently close to 50% (Figure 4). At the end of 4 weeks, participants reported on Likert scales (1=strongly agree to 7=strongly disagree) delivered by research assistants, which captured overall satisfaction, perceived likability, and pleasantness of the smartphone app. Data captured on paper-based survey forms were transcribed and stored in a secure database. As such, participants reported being satisfied (M_Satisfaction_=1.3) and expressed pleasantness and likability (M_Interface quality_=1.4).

A common qualitative finding from this trial included participants’ misunderstanding of the EMA questions, especially when they perceived a group of questions to be related to 1 another:

When I saw the “with anyone” question after the eating questions, I thought the app was asking me if I was eating with someone.

P30

Feedback from Field Trial 1 also included the need for balancing repetitive reminders versus interruptions that may be caused by an ongoing activity. Although several participants suggested resending an unanswered EMA question every few minutes as a reminder, a few discussed their inability to respond, irrespective of the reminder as they could not interrupt the ongoing activity:

It would help if the phone dinged after a couple of minutes to remind me in case I missed hearing the first time

P18

It won’t matter sending reminders really. My phone is not with me and I work in a warehouse with no network.

P20

As such, we identified an additional design constraint for the EMA system, namely *designing for increased engagement*. We hypothesized that engagement would increase from 2 additional auditory alerts on the mobile phone on receipt of an EMA question. That is, the phone will play an audio sound indicating that the first EMA question in a group is awaiting a response when a phone has received the question but when the user has not responded. In particular, the EMA system was programmed to sound an alert every 10 min if the mobile phone had an unanswered first EMA question in its notification drawer. As such, in Field Trial 2, we tested the hypothesis that sounding an alert every 10 min in a 30-min window of sending the first EMA question would increase the response rate as opposed to only sounding an alert once when the first EMA question is sent to the mobile device.

### Phase 4: Field Trial 2

This field trial included testing the hypothesis from Field Trial 1 for its effect on the response rate.

#### Methods

We used 2 versions, V3-simple and V3-ding, for use by 2 groups of participants. In all, 38 Healthy Me patients (P34-P72, Table 3) were randomly assigned to use either V3-simple or V3-ding for 4 weeks. During the 4-week field trial, both groups of participants received 12 EMA prompts daily, on the basis of their onboarding times. Similar to Field Trial 1, the actual EMA questions received by participants were dependent on their phone’s context or their response to the location question. All participants were instructed at study setup to consider each question independent of the other while responding, to overcome the confusion experienced by participants in Field Trial 1. Although participants using V3-simple received a single auditory alert, participants testing V3-ding received 2 additional auditory alerts when the first EMA question in a group was received by the mobile phone. The 2 sound alerts were spaced to occur at 10-min intervals when a participant did not respond. Both versions included the same group of questions seen by participants in Field Trial 1. This trial also included gathering qualitative feedback (semistructured interview or occasional phone conversations where the research assistants assisted participants during the trial when needed) on repeated notifications in addition to perceptions on question and response choices either at the end of the trial or during the trial at times when the participants needed assistance.

#### Results

We calculated the response rate using the formula identified in Field Trial 1. Response rates of the participants using V3-simple were compared between trials 1 and 2 by means of a linear model with the independent variable representing mean response rate of Trial 1 versus Trial 2. In this model, mean response rates of participants using V3-simple between Trials 1 and 2 were not significantly different (*P*=.16).

**Figure 5:**
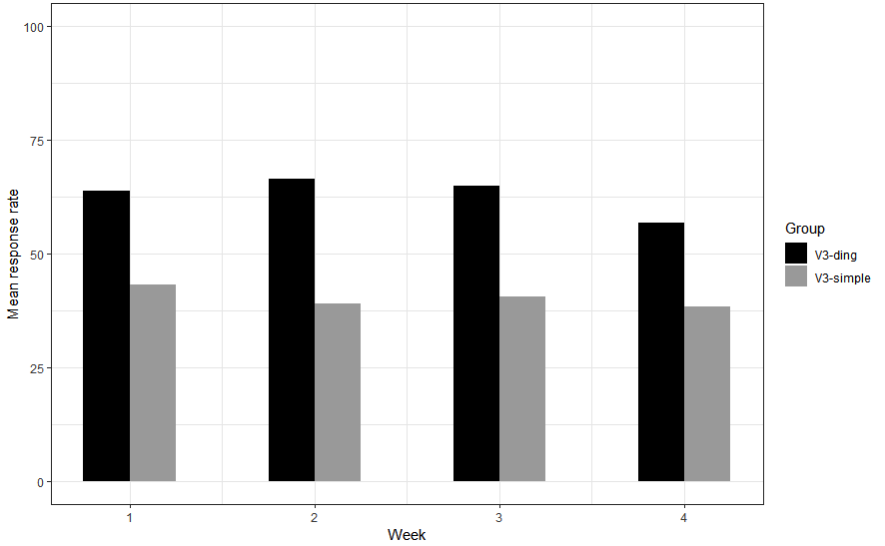
Chart depicting mean weekly response rate comparison between groups version 3 (V3)-simple and V3-ding for participants in Field Trial 2. V: version.

V3-simple is compared with V3-ding in Figure 5. We found a statistically and significantly higher response rate for V3-ding respondents. However, a greater percentage of participants worked in the V3-ding group as opposed to the percentage of participants in the V3-simple group (*P*=.03). Hence, the statistical test comparing the response rates between V3-simple and V3-ding groups in Field Trial 2 was estimated again with an adjustment for work status. This adjustment did not change the findings. There was a significantly higher response rate for participants in the V3-ding group than the V3-simple group (Table 4).

**Table 4:** Estimated means for response rate adjusted for group and work status.

Treatment	Estimate	Standard error	*P* value	Difference	95% CI
V3-simple	40.35	4.66	<.01	–19.96	–34.76 to –5.16
V3-ding	60.32	5.50	—^a^	—	—

^a^Not applicable.

The qualitative and quantitative findings pointed to a decline in the response rate with weekly progression. For instance, the qualitative reports on V3-ding indicated that some participants considered repeat sound alerts as nondisruptive whereas others perceived this feature as a drawback:

It is not a big deal.

P45

It felt like the system was eating up my phone’s battery.

P60

This feedback concurs with the occurrence of a statistical difference in the response rates between weeks 1 and 4 (*P*=.03).

Another common finding included participants being confused between whether they had to consider drinking water as a drinking activity:

Do I select yes when I have had water to drink?

P38

Water is a drink too. I reported yes every time I had just had water when I received eat question. I hope the system doesn’t think I am always eating or drinking something.

P42

## Discussion

Our aim in this research was to identify the constraints and design parameters for a mobile EMA system capable of capturing self-report measures of eating and movement behavior coupled with participants’ location and social contexts. We viewed the capture of these and similar health-related behaviors as they occur in everyday contexts as critical to progress in precision health research and interventions. Personalization of EMA processes may improve engagement in the data capture process and may also advance precision health efforts by facilitating just-in-time and just-in-place interventions [5]. By working with a diverse set of individuals, we were able to design an effective reporting interface. Following an iterative exploration and ideation process, we identified the constraints that users and stakeholders reported to be particularly important. We evaluated the interface and the self-report measures through 2 field trials. Although the first trial helped identify feasibility, the second trial tested a design feature that improved user engagement. Our interface was well received with high satisfaction and interface quality ratings.

Our target users were female patients with obesity seeking care in a safety-net health system; most had very limited household resources or technology experience and low health literacy. Achieving a satisfactory interface for real-world, real-time self-reports of weight-related behaviors and context required significant time with users and many design iterations and prototypes over a 6-month period. From this extensive effort, we suggest that future work attempting to conduct momentary assessments should achieve the following minimum specifications to support increased user engagement: (1) an onboarding process to personalize the times when an assessment is delivered to a participant, (2) a question pruning through passive sensing of device location to present only questions most relevant to a participant’s context, (3) increase participant’s attention with a notification that is prioritized over other app notifications in the receiving device, (4) limit the time window within which a participant can respond to a question to capture situations accompanying a behavior, and (5) repeat auditory alerts to remind participants to respond. Specific to reducing burden while capturing a user response, we suggest designing a system that (1) supports tap interaction to record a response, (2) uses simple-worded, direct questions with fewer words that are easier to read and quicker for the participant to understand, (3) has simple response options that are easier to read, quicker for the participant to understand and select from, and (4) includes instruction as to whether a participant has to consider each question in the assessment independent of one another while selecting a response.

### Our Contribution in Comparison With Previous Work

Although participants in our study used descriptions of the behavior similar to the findings reported here [28], we found that using icons to visually represent target behavior can be difficult to understand. Overall, we highlight that personalizing or tailoring the EMA experience to an individual can have several positive outcomes. First, customizing the times for EMA prompts to suit an individual’s daily routine can increase adherence. For instance, participants are more likely to not respond to an EMA question at times they do not want to be disturbed. Second, personalization can help to only present EMA questions that are most relevant to an individual’s situation or context. For instance, our system can automatically identify an individual’s location from the individual’s smartphone and present only the most relevant EMA questions. This can reduce the response burden and better match a respondent’s cognitive process for recall and self-assessment. Finally, our solution involving tap-to-report interaction highlights a design solution for reducing the response burden.

### Limitations

A potential side effect of EMA indicated in existing literature [29] is that EMA systems may serve as an intervention and not just a measurement tool. We heard some feedback supporting/suggesting that this may be the case. For instance, several participants expressed becoming more aware of their eating and movement behavior after using our EMA system. In other words, although participants were self-reporting eating or movement behavior, they were also being nudged to pay close attention to these self-behaviors. To determine to what extent this affected behavior or weight over time would require work outside of the scope of this report. Similarly, it is also outside the scope of this report to carry out validation of the EMA data. Future work could investigate the validity of EMA reports of movement, as this can be compared with accelerometry data. Social copresence could theoretically be validated by corroboration of the copresent individual. In the case of eating, validation would be more difficult as there is no objective measure of eating beyond direct observation; however, we do have work in the field using 24-hour dietary recall and EMA eating questions in the same time window. Future work can investigate a hybrid human-reported and automated data collection system such as the one described here [30] to support users to provide useful insights into their behavior at times when the automated system is inactive.

### Conclusions

With full appreciation of the potential limitations, we are intrigued by the possibilities of this EMA platform in 3 broad areas of future work. First, if asking an individual about a behavior frequently and in varied context has implications for the practice of that behavior, broad use of this EMA platform could result in an extraordinarily low-burden, low-cost, and highly scalable intervention tool. Thus, we believe the impact of the EMA reporting process on behavior, particularly weight-related behavior in our case, deserves careful investigation. Second, expanding this EMA platform to provide contextually appropriate feedback, whether human-reported or automated, is an interest of ours. Keeping to the design principles learned here while developing easy, simple, positively reinforcing feedback could enhance any effect EMA reporting may have on behavior. Third, the use of this tool to measure behavior and context for other projects and programs is significant as precision health advancements may well depend on EMA [31]. Examples in the literature point to exciting possibilities for work in smoking cessation [32], drug abuse and recovery [33-35], and mental health [36] to name a few. We are hopeful that ongoing work in these and other areas could lead to a new generation of behavioral data and interventions.

## Abbreviations

BMI: body mass index
EMA: ecological momentary assessment
FQHC: federally qualified health center
V1: version 1
V2: version 2
V3: version 3
